# A blockchain framework using proof of authority and smart contracts for ethical and secure healthcare asset management

**DOI:** 10.3389/fpubh.2025.1638546

**Published:** 2025-09-23

**Authors:** Muhammad Farooq Shaikh, Syed Hamza Hassan, Alessia Maccaro, Giacomo Pratesi, Davide Piaggio

**Affiliations:** 1 School of Engineering, University of Warwick, Coventry, United Kingdom; 2 Department of Computer Science, National University of Computer and Emerging Sciences (FAST-NUCES), Karachi, Sindh, Pakistan; 3 Department of Social Sciences, University of Naples Federico II, Napoli, Italy; 4 Paperbox Health S.R.L, Turin, Italy

**Keywords:** blockchain, healthcare, tracking, management, monitoring, asset

## Abstract

The proposed model in this paper focuses on asset tracking and monitoring in the healthcare industry and it uses blockchain technology. Data security incidents in the healthcare field have created financial and ethical problems over the last few years. During 2024, the money lost from healthcare data breaches often exceeded $4.88 million due to the exposure of private patients and asset information. This shows why it is important to use secure systems to protect worthwhile information and manage key assets efficiently and correctly. Since such intrusions cannot be prevented by the current systems, businesses end up losing money and working less efficiently. These issues are addressed by using a system that includes blockchain, IoT and digital asset tracking technologies. To ensure data integrity and prevent fake information, Proof of Authority (PoA) uses chosen, recognized authorities to verify and confirm each transaction. To protect transactions, PoA requires a number of approvals from different parties which helps ensure that transactions are safe and secure. With immutable and decentralized features, blockchain makes the management of assets more secure and transparent. Records of asset transfers and data are safely stored on the blockchain with smart contracts, providing real-time monitoring and no room for errors. When integrated with IoT devices, the system can constantly check all the assets, improving the company’s efficiency while reducing losses of items. The findings suggest that a PoA blockchain system can help healthcare asset management systems operate more ethically, safely, transparently, and efficiently.

## 1 Introduction

### 1.1 Background

Blockchain technology, used to ensure that information cannot be changed easily, has made it possible to build safe decentralized asset management and monitoring systems along with the consensus method (1). A system called Proof of Authority (PoA) works by using trusted members to check transactions, providing high security and fast transaction speeds without involving the time-consuming and power-hungry mining seen in mining-based systems (i.e., Proof-of-Work) (1). Additionally, the fact that blockchain cannot be altered or tampered with keeps it secure and provides a reliable structure for handling digital assets. In recent years, significant advancements in the field of blockchain have been achieved, which have demonstrated how it may improve security and privacy in a variety of sectors, including healthcare (1). For the healthcare industry, effective asset monitoring and tracking solutions are essential to ensure the integrity and security of key medical assets including, equipment, supplies, drugs, patients’ data, etc. While this is valid for all industries, the healthcare one is perhaps the most sensitive one, as security breaches would have a sensible impact financially and ethically wise. Just like technological progress, the evolution of cybersecurity threats is fast paced and ever evolving. For example, healthcare organizations reported a more than 29% increase in the number of incidents between 2020 and 2021. The cost of data breaches in 2020 and 2021 reached $7.13 million and $9.23 million resulting in substantial losses of various sensitive information like medical records financial transactions and patient identifiers together with financial expenses (2). International Business Machines Corporation (IBM) issued the latest data breach cost estimation showing 2024 stands at $4.88 million. The recent data breach estimates from International Business Machines Corporation (IBM) show a 10 % increase to reach $4.88 million which stands as the highest recorded amount thus far (3).

### 1.2 Economical and general data protection regulation (GDPR) factors

Healthcare asset management experiences economic impacts through higher production expenses combined with the possibility of GDPR non-compliance fines and negatively impacted patient wellness stemming from unavailable medical supplies and equipment during critical situations. Data breaches not only threaten a patient’s basic right to privacy but also compromise the accountability and auditability of critical healthcare assets, which are vulnerable to fraud and theft. Consequently, most systems may not follow proper asset tracking procedures, leading to delays or incorrect diagnosis and treatment of patients, posing a danger to patients’ safety and breaching data protection laws such as General Data Protection Regulation (GDPR). Sanctions that are applicable under the UK GDPR and EU GDPR include severe penalties such as fines of up to £17.5 million or €20 million, or 4% of global turnover, whichever is higher, for non-compliance (4). The reputation of healthcare institutions is at stake, and more importantly, patients’ lives may be compromised due to the sensitive nature of Healthcare Medical Records and Electronic Health Records (EHRs), which contain highly personal information (5).

Some efforts have been made to address security and privacy concerns in healthcare using administrative safeguards, physical safeguards, and technical safeguards, which form the pillars of Health Insurance Portability and Accountability Act (HIPAA) compliant protected health information security protocols. These protocols have been highly adapted. To further enhance privacy and security, organizations have mandated security awareness and anti-phishing training for all employees (6).

### 1.3 Significance of blockchain

Classic (state-of-the-art) approaches of managing healthcare assets like manual documentation and databases that are based on a centralized approach are vulnerable to issues relative to data loss, unauthorized access, and modification, which are the main factors behind cybercrimes (3–5, 7–11). The Health Insurance Portability Act (HIPAA) in the United States, for example, has a legal requirement that healthcare organizations take adequate data protection measures, yet frequently security breaches of sensitive patient information are reported such as medical history or treatment records (2, 3).

**Figure 1:**
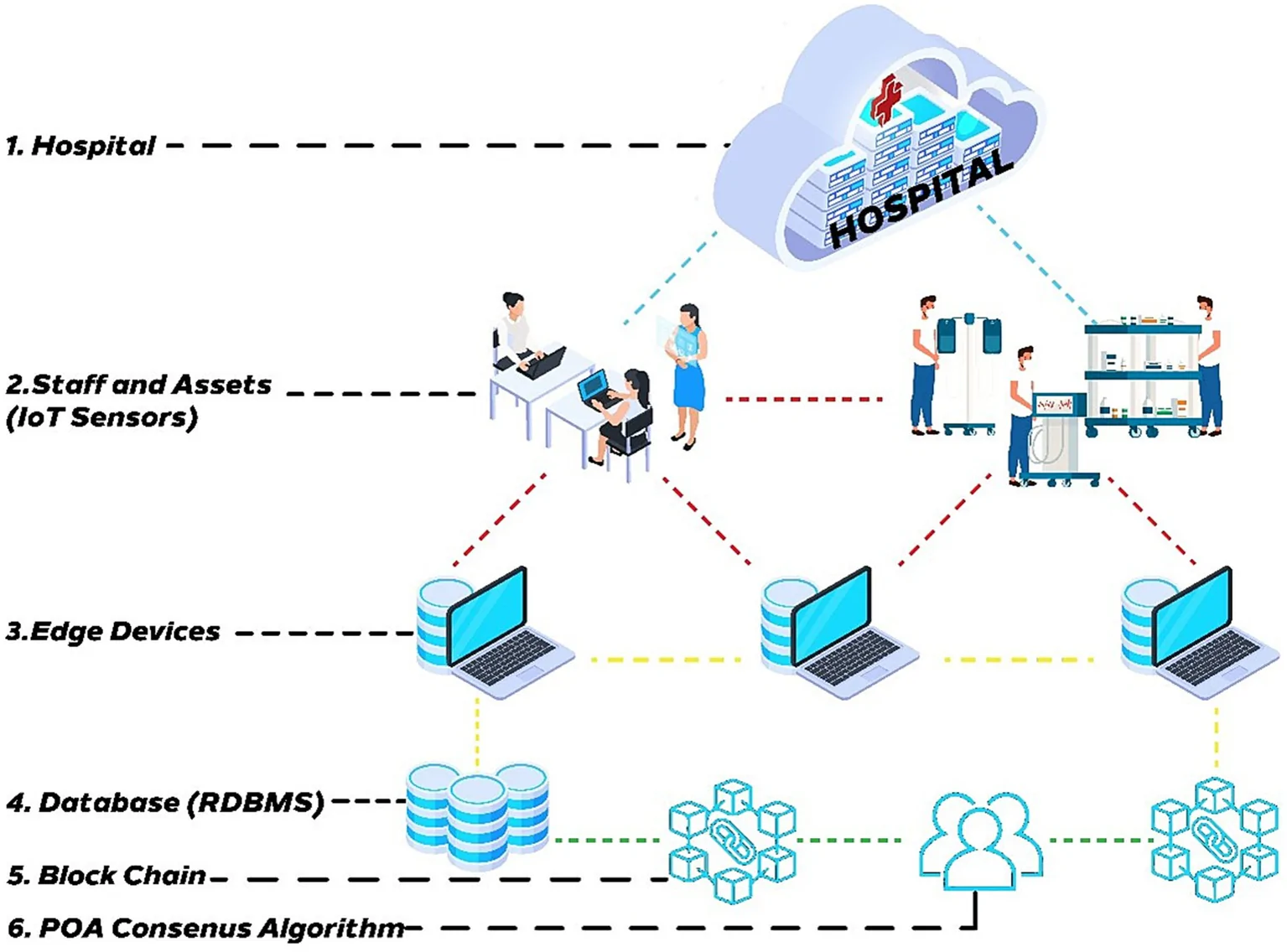
Complete visualization of permission-based blockchain framework in healthcare for asset management with PoA. RDBMS stands for “Relational Database Management System”.

On the other hand, blockchain is a highly secure, and immutable method of recording data on the blocks in the chain format. When incorporated with IoT-based medical devices, blockchain offers real-time tracking of assets, thus minimizing the effects of data loss, theft and fraudulent activities (5, 9–16). Furthermore, smart contracts are self-executing digital agreements where the terms are written directly into code. They automatically carry out actions, such as making payments or granting access to data, when specific conditions are met without needing a middleman. In healthcare, smart contracts can ensure only authorized people access sensitive information, helping with security and regulatory compliance (e.g., GDPR) because it limits the access of the data to specific people (9, 10, 12, 16). Hence, blockchain is a superior and currently the best option to implement instead of the classic frameworks, as it offers better possibilities to ensure data safety, traceability and compliance issues within the healthcare asset management (1, 8). Figure 1 presents the layout of a permission-based blockchain framework. One of its points of strength is its ability to ensure that once data is recorded, it cannot be altered, making the entire journey of an asset through the supply chain transparent and tamper-proof. Smart contracts (which are deployed on the blockchain network to provide automation for executing the specific set of tasks) also play a vital role by enforcing GDPR compliance, limiting access to sensitive patient and asset data to authorized personnel/administrations only. This reduces the chances of data leaks and ensures secure handling of healthcare resources (11, 12, 17).

**Figure 2:**
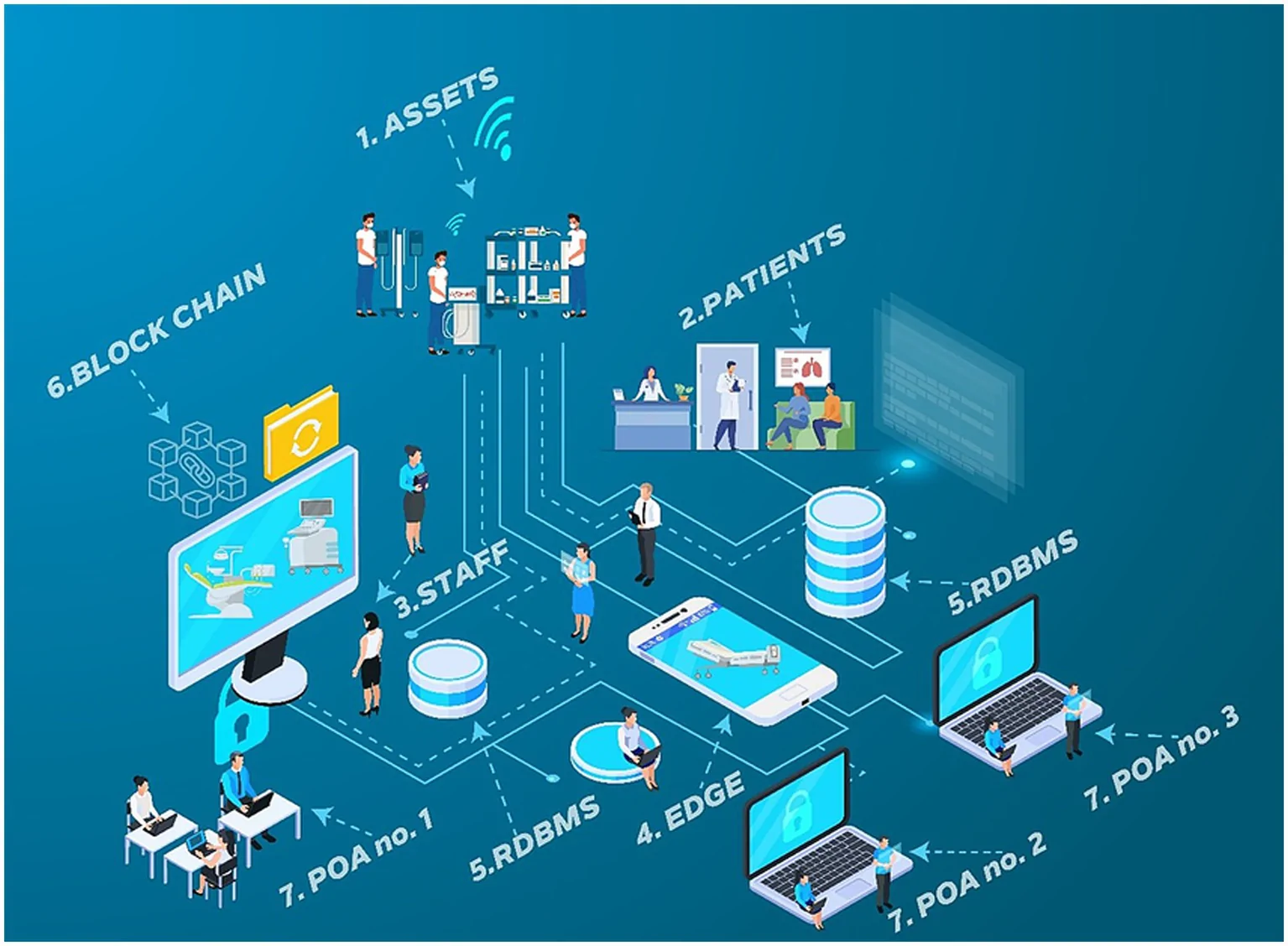
Flow diagram of the proposed blockchain framework with POA consensus for asset monitoring.

In the proposed framework (see Figure 2), a consensus mechanism based on PoA is employed, with three designated authorities. The reasoning behind this decision is that having just one authority poses too much risk, while two could result in a 50–50 tie, creating uncertainty. With three authorities, the system maintains balance, and this number can be increased if required to strengthen the decision-making process. By incorporating blockchain-based systems into healthcare asset management, the industry can improve data quality, transparency, trust, and accountability throughout the asset lifecycle.

### 1.4 POA-based blockchain

The transparency of blockchain technology offers real-time insight into the path taken by an asset, improving accountability and lowering the likelihood of asset loss or theft. Furthermore, by providing an auditable trail of all asset-related operations, streamlining regulatory reporting, and guaranteeing authenticity to the industry, blockchain provides effective auditing and authentic/secured monitoring. Thus, blockchain makes working together and transferring important information easier for stakeholders, making things work faster and easier while also using assets more effectively (18).

Blockchain solutions are facing difficulties when used to monitor and track assets in healthcare. Ensuring privacy and the safety of patients’ data requires considering ethical, legal and regulatory issues. Furthermore, building and deploying blockchain technology ought to account for the significant processing and setting up costs (like the fees for operations and transactions) (1, 6, 18).

In conclusion, a suggestion for how to make the most of blockchain in healthcare asset management is made here, with the aim to overcome difficulties, cut down costs and explore new ways it can be applied. This paper propose a novel framework which uses a PoA consensus mechanism with three appointed authorities. The framework is designed to profit from blockchains that have lower gas fees and cost less to operate and implement.

Finally, there are some improvements in healthcare logistics, one of the missing gaps is GDPR compliant, decentralized, transparent frameworks to ensure authority-based validation of assets tracking. Current blockchain applications are usually limited to EHR or drug supply chain management and miss clinical setting real-time and authenticated track assets (16–18). To fill that gap, the study proposes a safe, moral, and cheap blockchain-based asset-tracking system based on the Proof of Authority (PoA) consensus mechanism. The main goal is to illustrate how smart contracts on PoA can be used to monitor an asset transparently, auditable, and under the control of institutions.

## 2 Related work

### 2.1 Overview

This section explains in detail about studies on how healthcare is using blockchain for tracking and monitoring purposes. Man et al. (1) suggested using IoT-based healthcare asset monitoring systems (IoT-HAMS) that integrate ANNs and FL to help them perform better. By using information from medical equipment, they planned how many ventilators should be used, helping to distribute them wisely and making better use of resources during COVID-19. They claimed that making use of these models could reduce serious shortages because they allow planners to anticipate requirements. Additionally, the use of blockchain can improve the security of the predictive system. Blockchain allows all information to remain unchangeable which, along with digital twins, makes it easy to watch the data in real time and compare it to earlier estimates. This means that people working with the system have access to reliable data and machine forecasts to support decision making.

Mehta et al. (5) tried out a new system to guarantee complete traceability for assets as they move from one place to another in the supply chain. The use of timed activity records for each product allowed everyone involved, including clients, to confirm both the real status and positioning of every healthcare asset, handling transparency and accountability challenges in healthcare. In the same way, Tanwar et al. (11) created a blockchain framework for EHRs to make patient information more secure, showing improvements in privacy and access restrictions. Rouhani et al. proposed MediChain™ as a secured, permissioned blockchain solution for sharing medical information. Although the system makes assets safer, it does not have a well-defined way for networks to be led. To address this challenge, we suggest using a PoA consensus method where important members of the network confirm every transaction. As a result, there is tighter management and better defense against fraud and tampering, unlike in MediChain™, where authority is not clearly assigned. Demircan-Yıldız et al. (18) analyzed the logistics in hospitals and found that around 35% of a staff member’s effort is wasted on locating mobile property (movable medical equipment such as ventilators, infusion pumps, wheelchairs, diagnostic equipment, and all other transportation resources essential in providing patient care) leaving a hospital to lose up to $1 million each year. To overcome this, they stated that a solution based on real-time monitoring, discrete event simulations, and multi-objective optimization should be used. This could be improved by adding blockchain technology to ensure records of assets are always reliable and seen by the intended parties only.

Simultaneously, using federated learning (FL) in combination with blockchain is helping to solve ethical and practical issues in the healthcare sector. According to Qu et al. (19), applying blockchain to FL in collaborative learning systems strengthens their decentralization, trust, and member control. Zero-Knowledge Proofs (ZKP) and Distributed Ledger Technology (DLT) are some of the mechanisms used by FLchain (Federated Learning + Blockchain) frameworks (20, 21) to guarantee security, data privacy, traceability, and integrity in collaborative learning. In addition, many FL-blockchain (22–24) handle adversarial threats, power-saving properties, and ensuring all users are included. Specifically, Blockchain-based Federated Learning with Committee consensus (BFLC) [25] connects the FL approach with committee voting to check rouge validators and keep blockchains scalable and well-spread. Although integrating Federated Learning (FL) and blockchain shows conceptual promise in terms of privacy-preserving analytics, this approach becomes, by and large, an experimental one in the case of healthcare. The main practical deployment challenges are the heterogeneity of data held in different institutions, the limits of computation at the edge, the costs of synchronization, and the absence of standard toolchains. Therefore, our mention of FL-based systems is hypothetical and points to future possibilities instead of comprising part of the adopted system. In conclusion, such hybrid systems outline a path forward for managing assets by ensuring integrity, ensuring honesty, and making quick and reliable decisions when systems are not centralized.

### 2.2 Summary and analysis

According to Kakarlapudi et al. (15), to fix the obstacles they saw in the literature, new solutions should be created, including updates to consensus algorithms, introducing compatible smart contracts, and using Web3 tools so that different platforms can work well together.

Both Mehta et al. (5) and Kakarlapudi et al. (15) mention that blockchain improves data integrity, visibility, and how private data is managed. However, they do not sufficiently explain the problems of having to pay high transaction fees and bear the high costs of keeping public blockchains running. The problems mentioned can be reduced by relying on networks like Ethereum or Binance Chain, since they are more efficient and cost less. In addition, using PoA smart contracts can help ensure that the GDPR is being followed.

To solve the problems of asset inefficiency identified by Demircan-Yıldız et al. (18), our blockchain framework requires medical managers to grant permission before assets can be moved. By doing this, the administration can track the use of resources and ensure that no one is favored more.

In addition, federated blockchain architectures, as mentioned in (19–24), help with distributed learning, provide secure ways to train data models, and resist threats from adversarial data. This makes it possible to use privacy-protected asset tracking in real-world healthcare situations.

## 3 Methodology and system implementation

The section presents a plan for using blockchain technology to keep track of assets in healthcare. It is built using the existing ERC-20 network and includes web3.js libraries. The framework adds PoA authentication feature to confirm and protect the mobile assets. The web-based application prototype corresponds to the ERC-20 test net smart contracts that operate in a decentralized manner. The web3.js-based web application connects directly to the ERC-20 network using MetaMask.

### 3.1 PoA-based blockchain framework

The proposed framework adaptation in the real-world scenario is the main goal of the implementation phase. There are numerous crucial milestones in this phase.

### 3.2 Design and development of the framework

Based on the proposed architecture, the blockchain-based framework is created. This involves creating smart contracts including the PoA authentication system. To deploy PoA, a testnet of three accounts as validators was used focusing on healthcare authority representatives. These validators act like actual settings of hospitals or regulators. The validation of transactions was fulfilled by a majority signature (i.e., 2 out of 3). This proposed approach employs three Proof of Authority (PoA) design to achieve the equilibrium between security, efficiency, and cost. A single validator (1-of-1) would mean a single point of failure and therefore compromise on the idea of decentralized trust. On the contrary, a two-validator (2-of-2) setting is subjected to deadlock in case of disagreement and this leads to stagnation of transactions. The authority of the three authorities allows the system to have a majority (2-of-3) that is always achievable, eliminating the possibility of ties and enhancing reliability. Although increasing the number of validators to above three would boost fault tolerance and decentralization, it would boost the computational cost and signature verification process and gas consumption. Therefore, the worst case scenario and the optimum starting point was chosen to be three since it has resilience and integrity without excess baggage. The model is also scaleable and more validators may be introduced in case more robustness is necessary in practical applications.

Furthermore, the framework was created with Solidity-based smart contracts deployed to a local Ethereum testnet operating on Ganache. The front-end interface was developed on React.js, and the server-side communication using PHP. Web3.js APIs were utilized to interface the Ethereum blockchain to allow efficient communication of the smart contracts with the user interface. The MetaMask browser extension initiated and signed transactions, and was the user-side wallet and interface through which blockchain confirmation occurred. During the development and initial deployment of the contracts, Remix IDE was applied to estimate gas, debugging, etc. Moreover, any communication with asset data (e.g., registration, status update etc) was performed using HTTP POST/GET requests and these were logged. Finally, all of this infrastructure can be configured with Ganache, MetaMask, Web3.js, Node.js, and PHP, along with the open-source smart contracts and UI components included in this project.

### 3.3 Integration with IoT sensors and APIs

**Figure 3:**
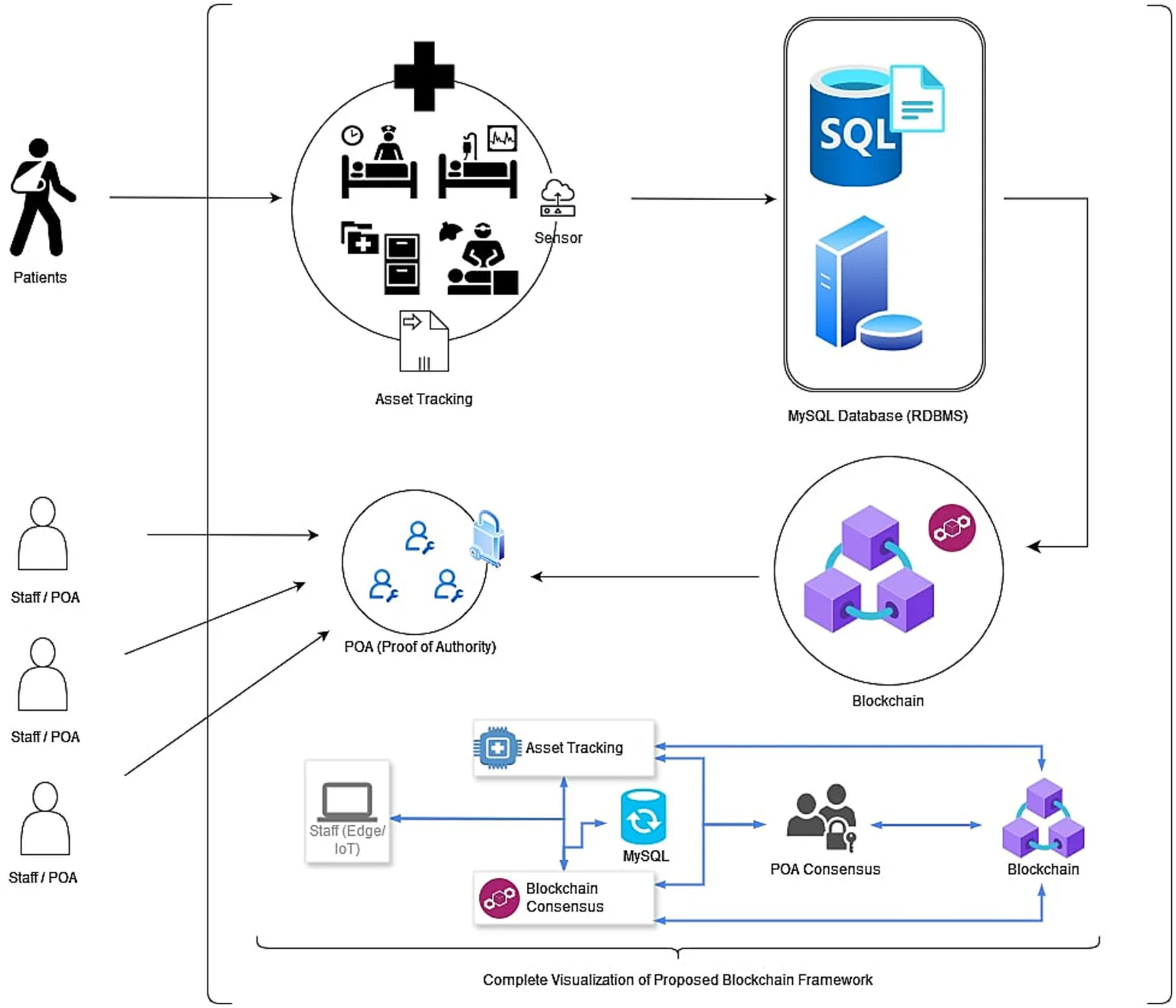
UML inspired diagram with a complete visualization of proposed blockchain framework with smart contracts and POA.

In real-world situations, information is gathered through IoT devices that continuously transmit location data. Real-time asset monitoring is made possible by integrating IoT sensor data with the web-based application via APIs (Get and Post) and gateways. The proposed blockchain-based framework for security healthcare asset tracking with data management features is presented in Figure 3. All framework elements consist of patients together with healthcare staff acting as Proof of Authority validators and separate asset tracking and central MySQL database components. The main transactions between blockchain nodes get synchronized directly to the blockchain ledger to protect records from tampering. Furthermore, asset tracking device data gets stored simultaneously within the SQL database and the blockchain system. The Proof of Authority (PoA) consensus mechanism authorizes designated personnel to validate system transactions along with maintaining trust throughout the network. Moreover, the data flow operational part of the figure shows how the asset tracking layer uses MySQL and blockchain components for transparent secure real-time healthcare resource management. Finally, data integrity with enhanced traceability and strict accountability features distinguishes this architecture when operating in sensitive healthcare settings.

### 3.4 Asset tracking data schema

**Figure 4:**
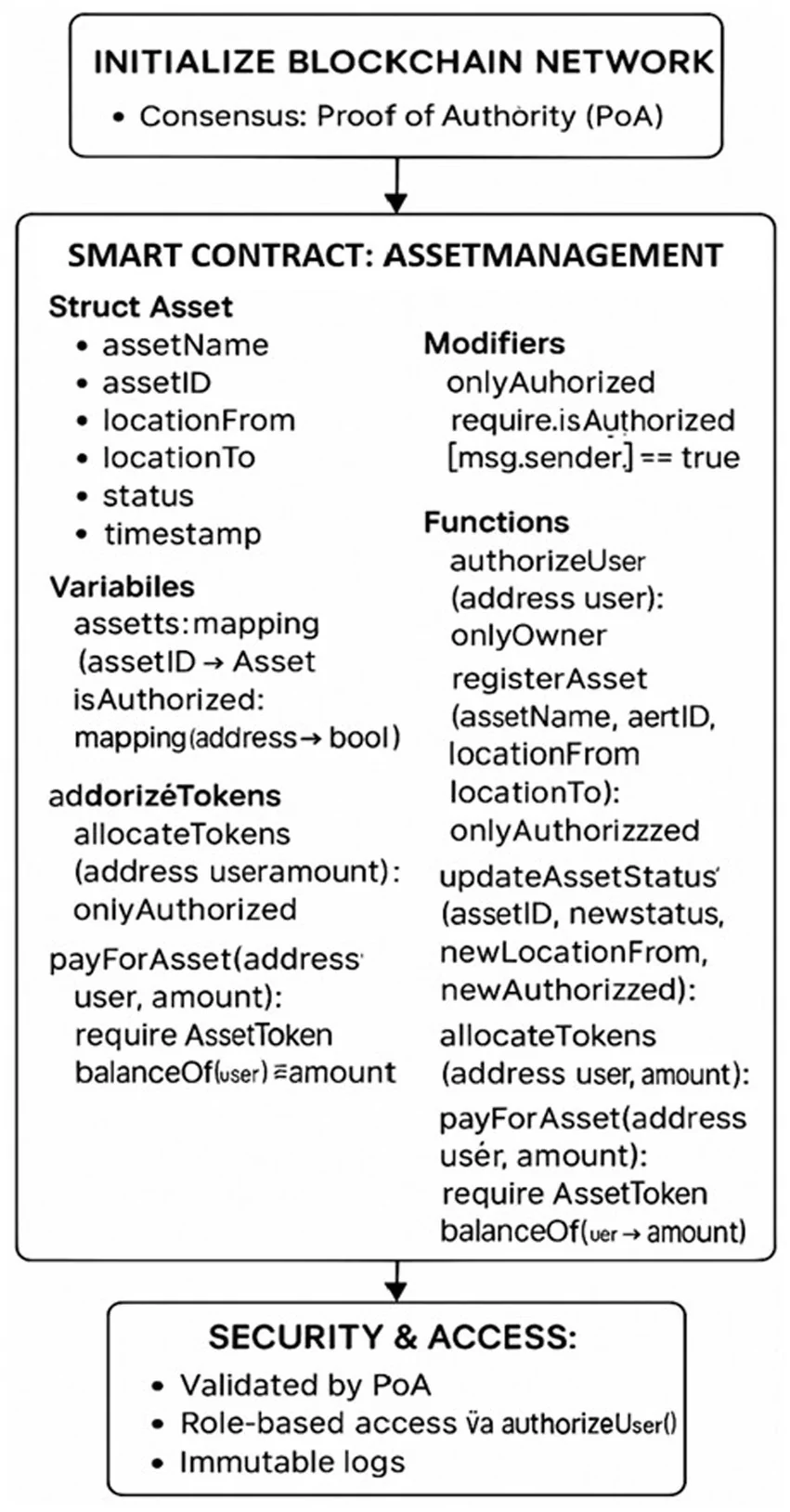
Flowchart of a blockchain-based asset management framework using proof of authority (PoA) and ERC-20 smart contracts—includes asset registration, status updates, token transactions, and access control mechanisms.

The parameters used in this framework’s sample data collection, as shown in Figure 4, are designed to ensure comprehensive asset tracking and monitoring. The User ID (U-ID) represents a unique identifier for each user interacting with the system. Task denotes the specific action being tracked, such as asset management, tracking, or monitoring. The Moved From and Moved To fields capture the origin and destination of the asset during the tracking process. Asset ID is the unique identifier assigned to each asset, while Owner stores the transaction hash of the person or entity responsible for initiating the action. Status indicates the current state of the transaction within the Proof of Authority (POA) consensus, showing whether it is pending, in a tie, successful, or unsuccessful. Next Level refers to the current approval stage in the POA system, with three levels (1/3, 2/3, and 3/3) representing the approval progress. Finally, “Actions” show whether the transaction is pending or completed. These parameters collectively provide a structured and transparent tracking system for monitoring assets throughout their lifecycle in the blockchain framework.

### 3.5 Smart contract development and deployment

To simplify asset tracking and monitoring, the appropriate smart contracts are created (pseudocode can be seen in Figure 4 in a flow chart diagram). To provide the highest level of security and immutability for the recorded transactions, these contracts are created on the decentralized ERC-20 blockchain network. However, sensitive information is not kept directly on the blockchain to respect privacy laws in the present version of the project. Examples of this are asset IDs, transaction hash identifiers, wallet addresses, recent changes in status and recordings of events such as registrations or updates. Off-chain storage is used for sensitive information such as personal medical records to ensure the data is private and compliant with GDPR.

### 3.6 Pseudocode (flow chart)

**Figure 5:**
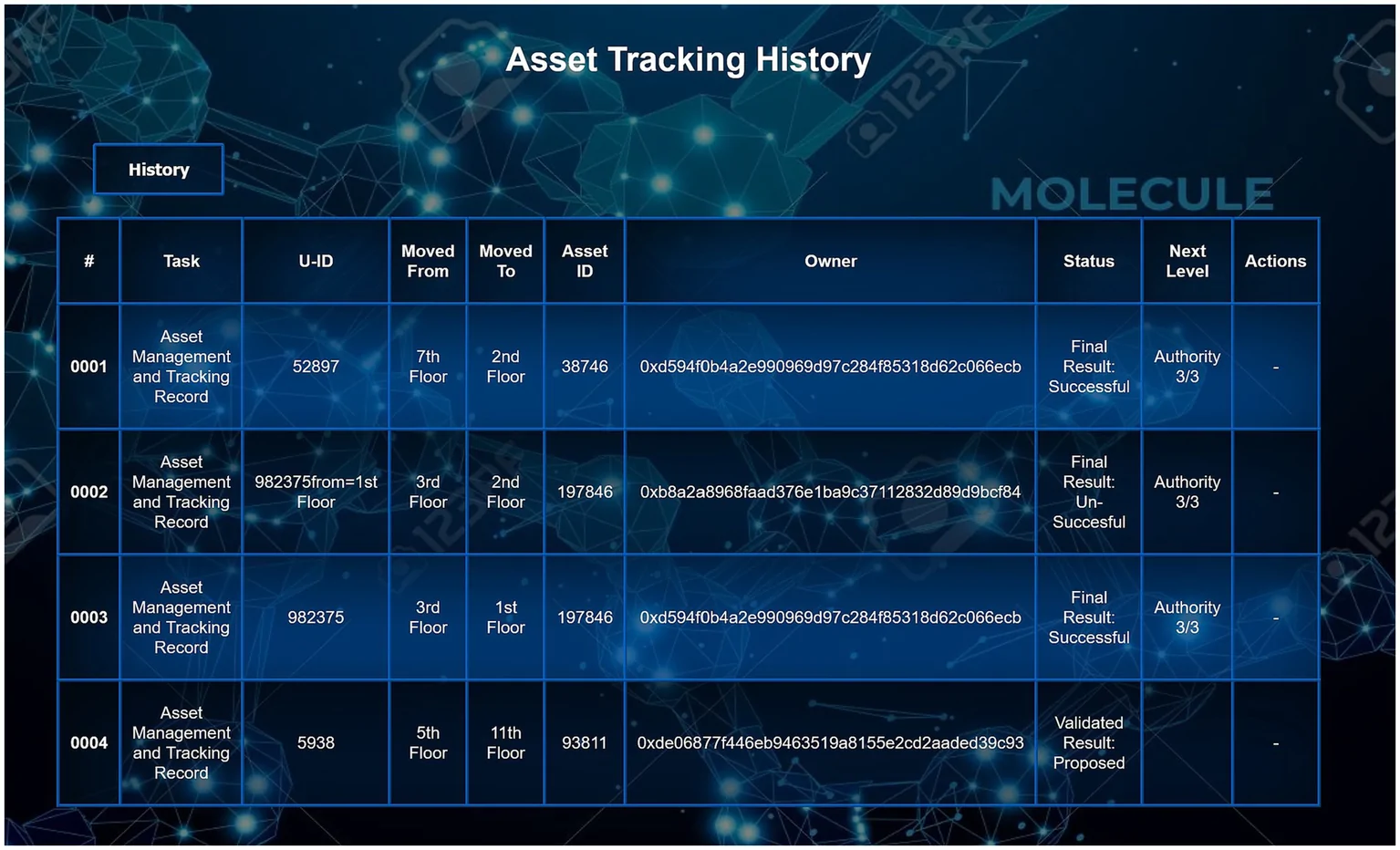
Blockchain-based asset history interface showing traceable logs of medical equipment, including asset ID, owner, status, and validation details, enabling full transparency and auditability.

**Figure 6:**
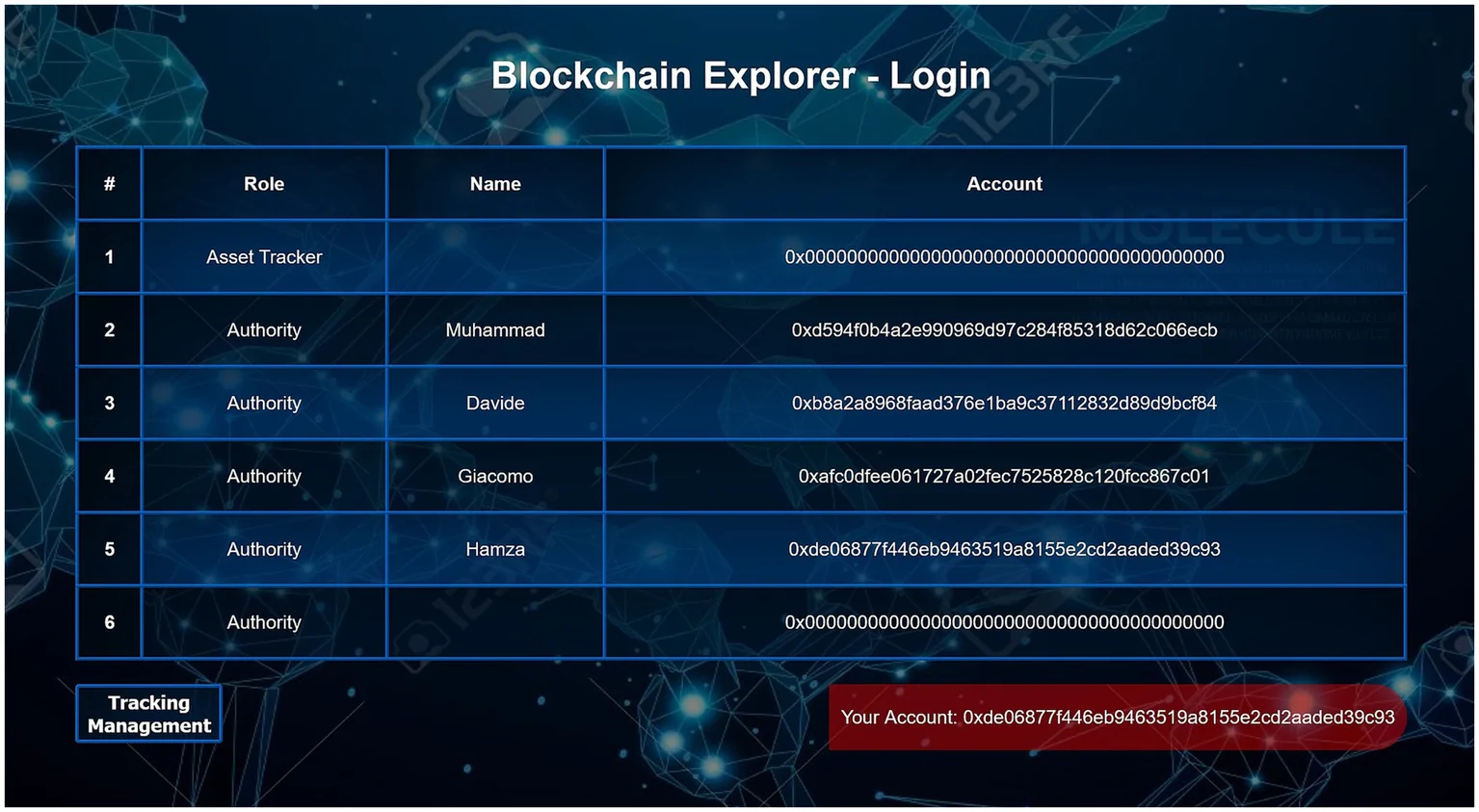
Blockchain network explorer displaying participant roles, identities, and associated public keys, emphasizing secure access control and role-based permission management using Proof of Authority.

The following pseudocode and flowchart representations (Figures 4–6) illustrate the operational logic and structure of the proposed asset tracking system using blockchain.

### 3.7 MetaMask wallet integration with web application

As shown in Figures 5, 6 to connect to the ERC-20 network, the MetaMask wallet is incorporated into the web application using web3.js. Secure interactions and transactions with the blockchain are made possible by this connection. Furthermore, Figures 5, 6 present the interface of a single asset and the set of roles assigned to each participant in the system. Figure 5 illustrates the chain of every asset transfer and Figure 6 points out the management of identities and permissions using the PoA algorithm to assure the system’s security and organization.

### 3.8 Verification and validation

In this phase, the proposed structure is tested and evaluated using supervised executions and distributed transaction tracking by network computers. Various methods were studied for collecting data, including simulation, connecting to real IoT systems and examining sample datasets. This paper used a sample-based approach which involved making simulated data to study asset management and tracking. It allowed easy testing of how fast the framework ran in a stable environment. Although randomized simulation inputs and IoT streams are good alternatives, choosing a sample helps with predictable and reliable testing. Furthermore, by integrating the data through API calls in both the GET and POST ways, the user interface and the blockchain backend could interact in real time. Since the data was provided, ERC-20 smart contracts were used to guide the transactions for tracking assets. Furthermore, all transactions were safely kept on the blockchain, letting them be checked and verified whenever necessary. The method used the Proof of Authority (PoA) algorithm which required three digital confirmations from designated authorities for each transaction to be valid. The system was designed in a way that made unauthorized data changes unlikely. Once validation was complete, all details of every transaction were saved to the ledger, meaning the asset monitoring information remained truthful and steady in the system.

### 3.9 Results gathering

This phase is dedicated to studying and evaluating the designed asset tracking system for blockchain technology. It involves checking whether the framework works well and whether its smart contracts are executed properly, the authentication system is safe and there is no inconsistency in on-chain records. The tracking accuracy of the system is examined by comparing the data stored on the blockchain with the actual positions and movement of assets. The framework is also assessed next to other asset monitoring products to stress its special capabilities and improvements. This evaluation makes clear that the use of Proof of Authority (PoA) gives added assurance, gas usage is more efficient, and the records cannot be changed, meaning the system is well-suited for stable and safe use in healthcare.

### 3.10 Indicative gas costs of key actions

Table 1 below provides some insight into how much gas will be required for key smart contract tasks according to the proposed framework, based on recent gas usage and prices. The estimates reveal how such actions on a public blockchain could affect the financial situation. However, gas costs and ETH-to-USD rates change a lot and frequently, based on the state of the market and network pressure. Therefore, the costs for executing transactions may differ in different countries at different times. Data on gas fees was obtained directly from the Etherscan Gas Tracker.^1^

**Table 1:** Indicative Gas Costs for Key Smart Contract Functions on the Ethereum Network.

Smart contract function	Gas estimate (Gwei)	Estimated cost (USD)*
REGISTERASSET()	151,213	~$0.71
UPDATEASSETSTATUS()	124,374	~$0.58
AUTHORIZEUSER()	60,000	~$0.28
ALLOCATETOKENS()	151,168	~$0.71

*The estimation assumes that the price of gas is 1 Gwei (1 Gwei = 0.000000001 ETH), and Ethereum market value at 13 August 2025, 4687.46 USD/ ETH (source: Google Finance).

The ETH cost can be calculated by means of the formula:


GasUsed(Gwei)×(1ETH/1,000,000,000Gwei)×ETH−to−USD

Example:


151,213×(1ETH/1,000,000,000)×4687.46≈$0.71

**Figure 7:**
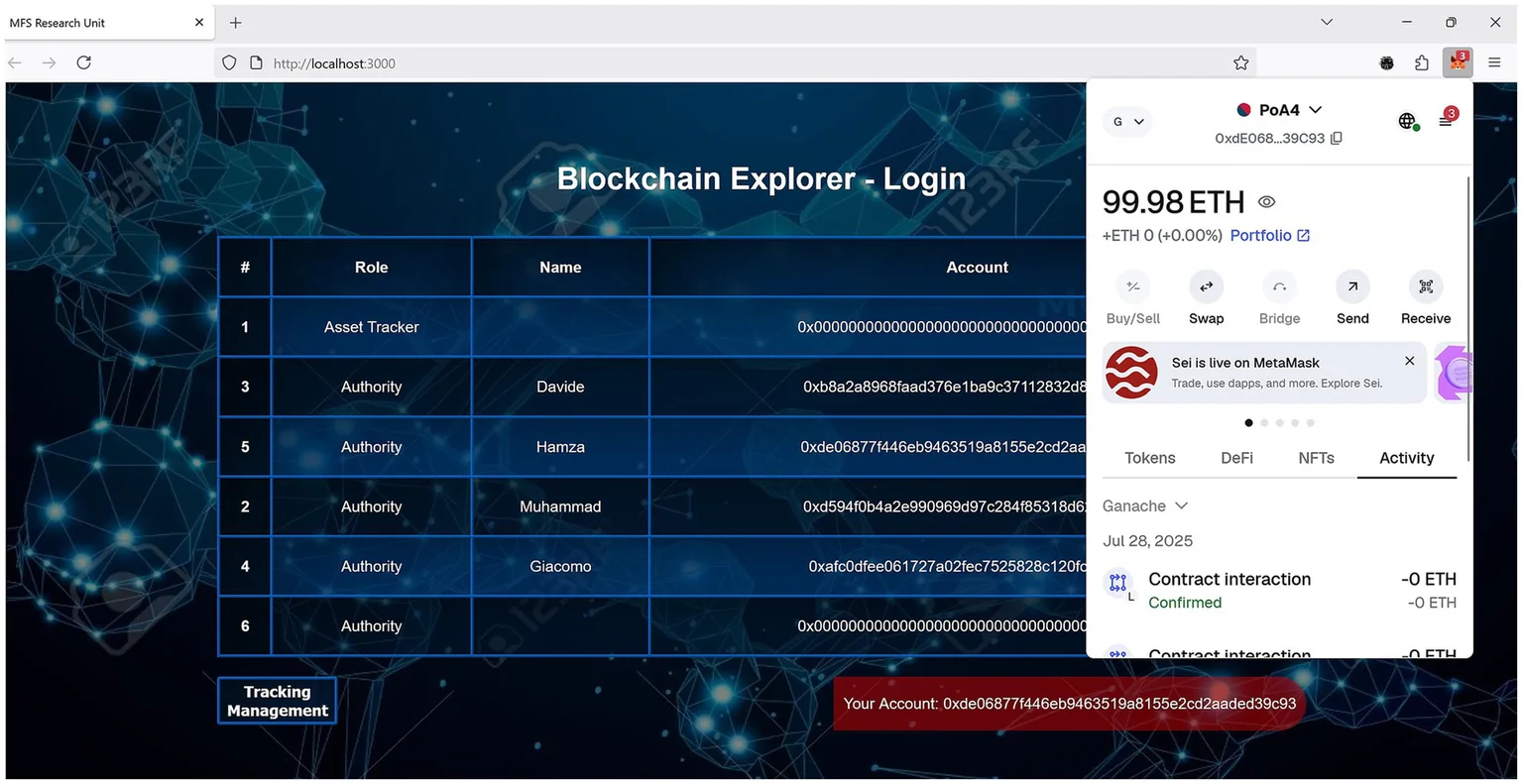
MetaMask browser extension connected to Ganache testnet browser snapshot with user wallet availability and simulated ETH balance and contract interactions history display. This checks testnet connections and account-based access confirmation.

**Figure 8:**
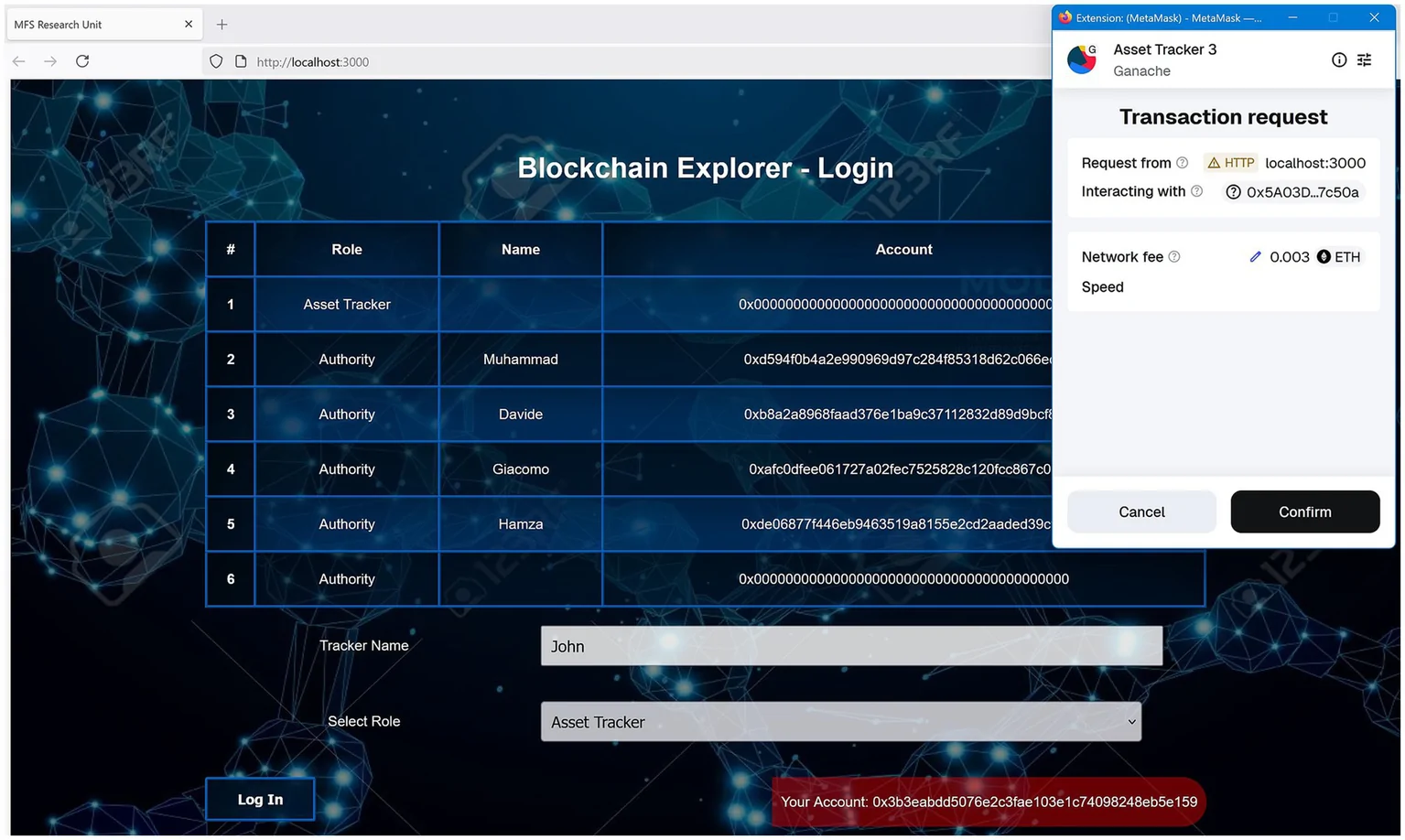
The request of the transaction was completed via the local DApp (http://localhost:3000), which asked MetaMask to confirm the transaction. The network shown fee of 0.003 ETH shows an actual time estimation of the gas cost upon running smart contracts.

**Figure 9:**
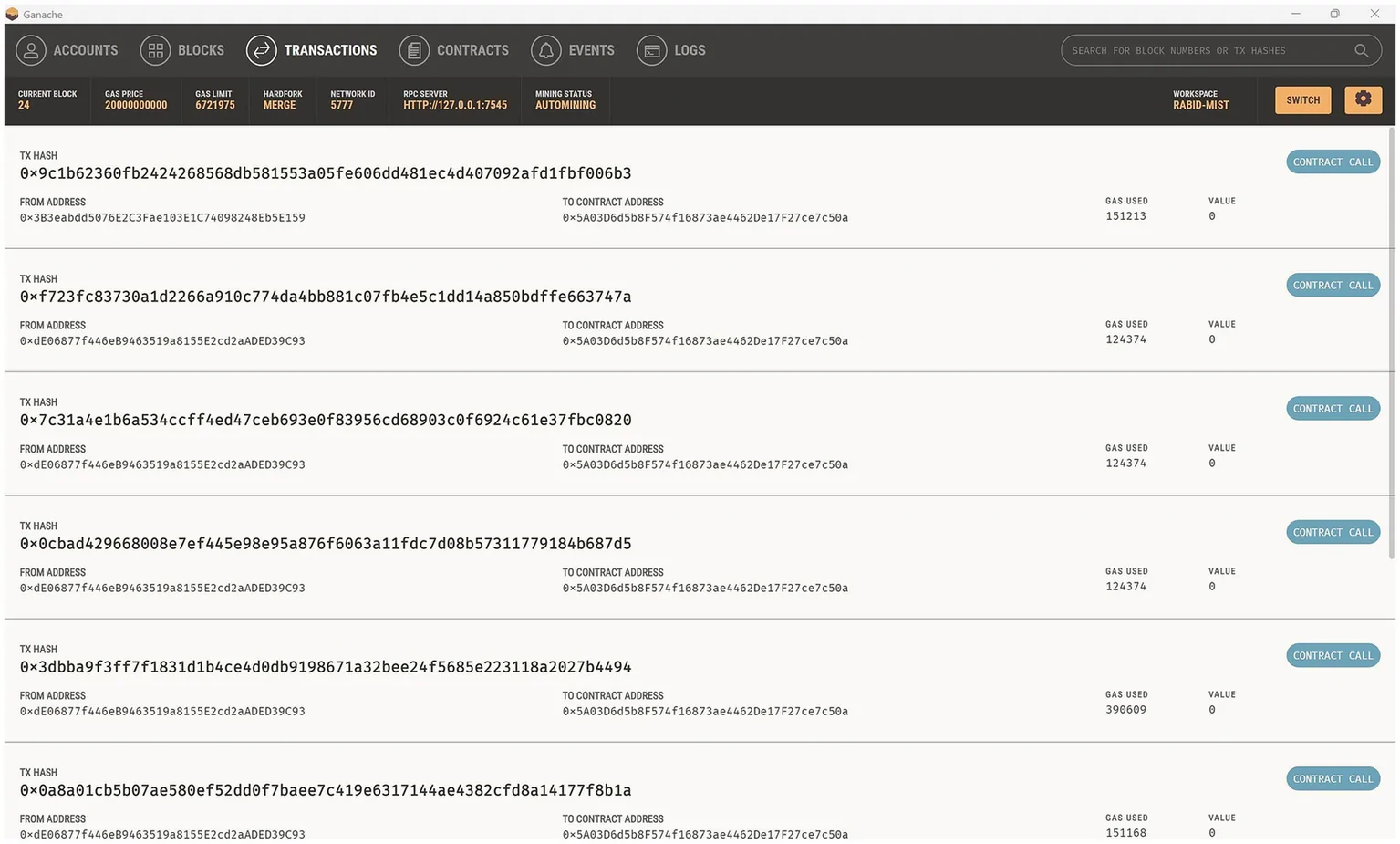
Ganache UI display of a listing of contract invocations and these were actual gas used per transaction, validating that a number of smart contract functions were called and mined successfully including registerAsset() and updateStatus() calls.

**Figure 10:**
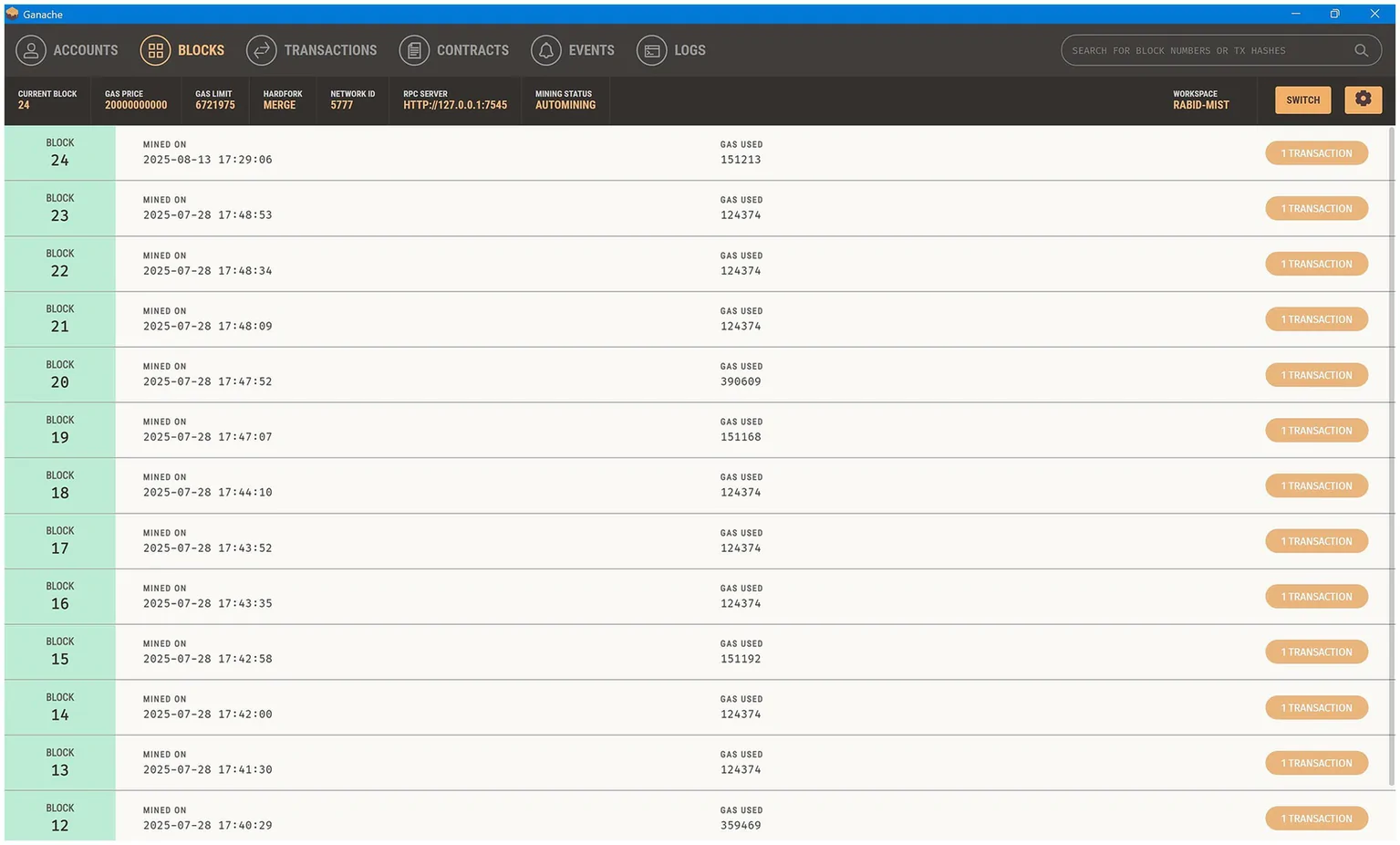
Mined blocks overview of the Ganache testnet. Each block identifies the amount of actual gas that the transaction it encloses consumes, which confirms the used gas estimates in the performance analysis section.

To validate these estimates, screenshots of our test deployment on the Ganache testnet and MetaMask interface demonstrating the real-time execution and gas usage can be seen below:

The above screenshots (Figures 7–10) offer visual support that the framework worked on a synthetic blockchain environment. The wallet functionality (including its integration and signing of transactions) was implemented using MetaMask, and Ganache was used to serve a local Ethereum testnet. The gas consumption indicated on MetaMask and Ganache logs validates realism and testable execution, which enhances the accountability of performance parameters presented in this paper.

## 4 Results and discussion

### 4.1 Results

**Figure 11:**
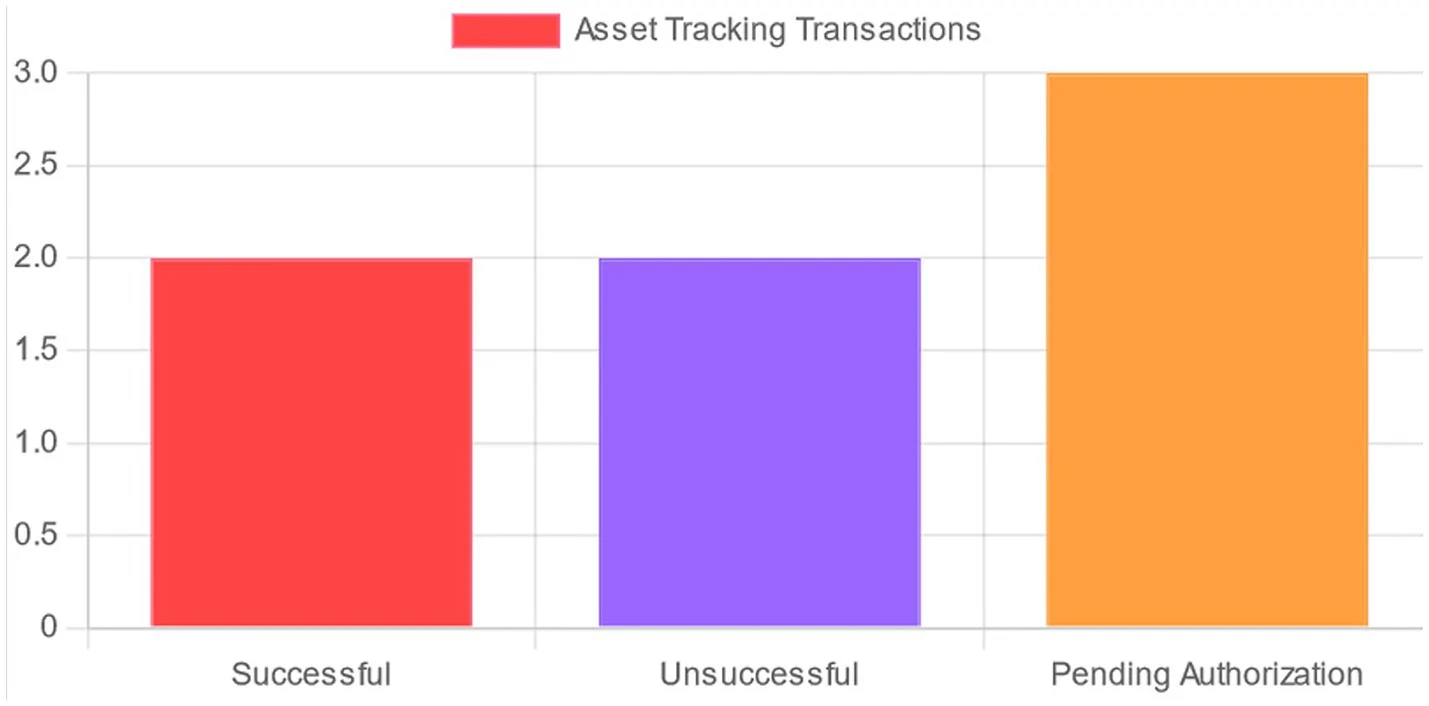
Results & Findings of all transactions being written in the proposed blockchain framework. The chart highlights the bare-bones success, failed, and pending asset tracking transactions.

The chart shown in Figure 11 displays the partitioning of asset tracking transactions in the blockchain infrastructure. The graph displays different types of transactions using different colors. Successful transactions are represented in red, unsuccessful operations are marked in purple and pending transactions are shown in orange. The red bars represent successfully authenticated and valid actions with the smart contract. Finally, the purple bars denote failed transactions, pointing to the importance of access control in ensuring the security of the system. Orange bars represent the largest number of transactions requiring validation from validators using the Proof of Authority (PoA) mechanism. Furthermore, a backlog at the validator level could indicate a way to improve performance. The chart expresses that the system has some authorization issues that could be resolved for enhanced performance.

### 4.2 Discussion and Findings

This paper presented a blockchain-based asset tracking framework designed for secure, transparent, and efficient monitoring of healthcare assets. The combination of ERC-20 and PoA ensures that the environment offers secure access, validated data and permanent record-keeping for all transactions. Furthermore, a successful track record of using the blockchain framework shows that it is a reliable and efficient method for asset management in healthcare. The system’s authentic actions prove that it reliably records and keeps all transaction details using the Proof of Authority (PoA) system. Figure 11 illustrates that keeping transactions consistent helps maintain the framework’s reliability. Moreover, the findings show promise, but they are limited since the evaluation uses samples instead of data experienced in the real world. Furthermore, occasional hang-ups in transaction confirmations highlight the key role that validators play in proof-of-authority networks.

**Figure 12:**
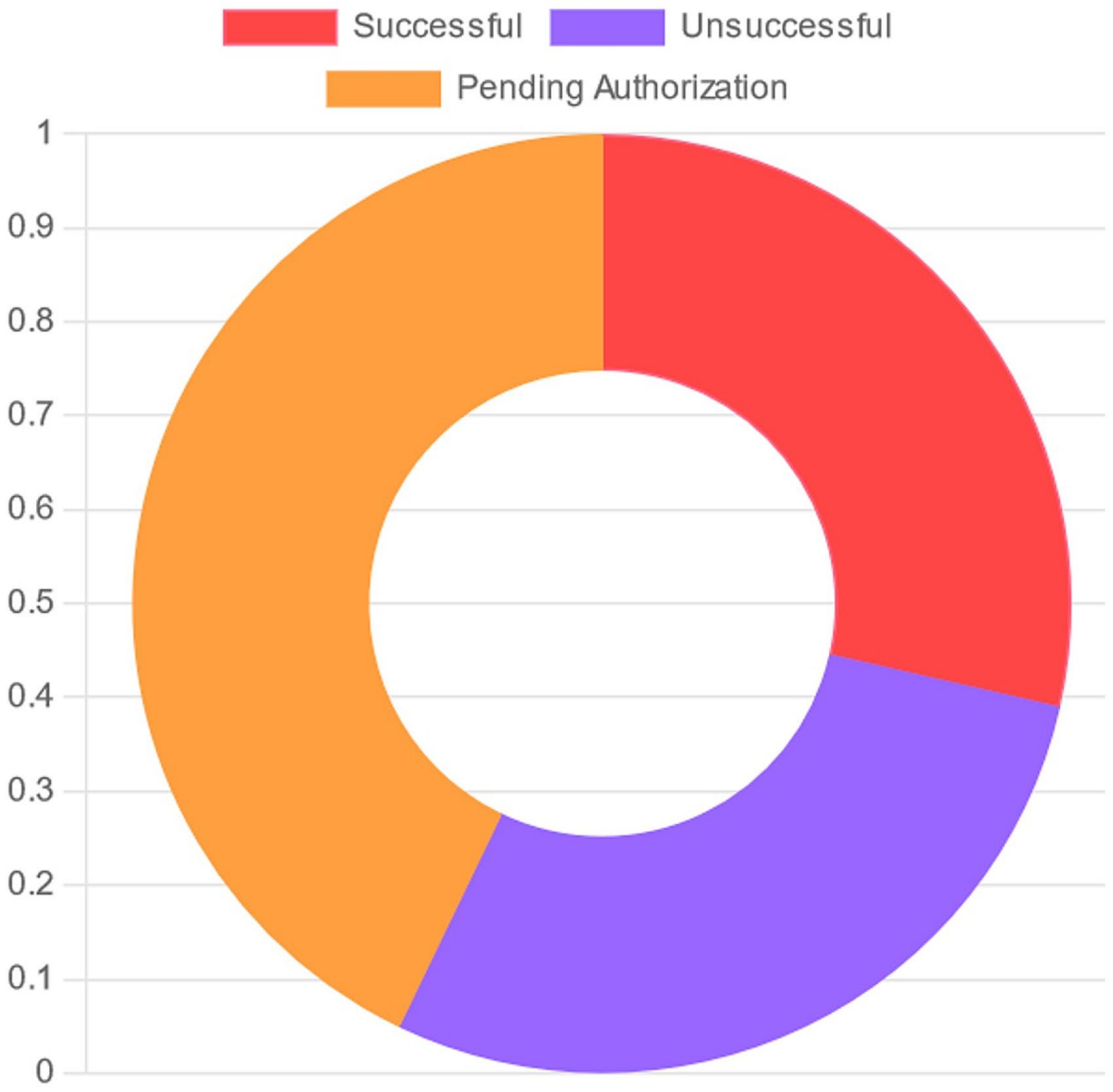
Distribution of asset tracking of transaction types in proportional in the block-chain Framework. The given perspective allows analyzing the percent breakdown of each type of transactions, which provides a comparative picture of systemic throughput and bottlenecks.

Where Figure 11 points out absolute values of transactions, Figure 12 above illustrates a cumulative distributional review. The incorporation of the two increases the capacity to understand the performance of the system in both operational and strategic ways. Furthermore, Figure 12 illustrates a donut chart representing how the different transactions relating to asset tracking are spread across different processes within the blockchain system. Each bar in the chart represents the proportion of transactions in relation to the total number of events occurring within the system, with 1 indicating the maximum value. It allows the user to understand the relative importance of each transaction type in the complete framework. The categories represented within the chart are Successful (shown in red), Unsuccessful (displayed in purple) and Pending Authorization (presented in orange). This diagram (Figure 12) illustrates how each category contributes to the system proportionately. The chart illustrates that most transactions are pending approval, indicating many transactions that have yet to be validated. The remaining areas of the chart are devoted to illustrating the occurrence of completed transactions and instances where validation has failed. Unlike the previous Figure 11 comparing the exact numbers of transactions in each category, this chart shows how each category is distributed in relation to the others. Instead of highlighting quantity, this chart helps assess the overall health and efficiency of the system by revealing disproportions between categories. This chart adds value by offering a unique way to look at the system. It allows for rapid identification of any potential bottlenecks within the system. Both graphs contribute to a more comprehensive understanding of how assets are managed through the blockchain.

### 4.3 Limitation

Since the research is neither experimental nor measurement based, there are no traditional limits of detection or quantification that can be applied. Nonetheless, the validity of transactions was also determined within the definition of the smart contract logic through PoA validation thresholds (e.g., 2-of-3 validator confirmation).

## 5 Ethical considerations

Data breaches in the healthcare sector raise significant ethical concerns, as they compromise the fundamental right to privacy and patient self-determination. The principle of respect for human dignity requires that personal health information be handled with the highest level of security and confidentiality. However, the vulnerability of existing systems endangers the trust between patients and healthcare institutions, undermining the doctor-patient relationship and the quality of care provided (26). Another crucial ethical aspect concerns distributive justice: the lack of adequate protection and traceability measures for healthcare assets can lead to inefficient resource distribution, disadvantaging the most vulnerable patients. Delays in the delivery of medications or medical equipment can cause irreversible harm, violating the principle of equity in access to care (25). The proposed blockchain-based framework that uses artificial intelligence and adopts the Proof of Authority consensus method allows healthcare organizations to keep track of and govern their important assets and sensitive patients’ data in a safe way. Moreover, authorizing transactions using permissioned keys makes it easy to locate and hold responsible anyone who misuses or makes use of healthcare equipment without authority.

Although blockchain and AI can help protect healthcare data, their use should always be carefully considered based on what is ethical. Though they increase both security and transparency, they bring new problems related to tracking, privacy and other organizations owning your data. Consequently, employing technology in healthcare must be balanced, respecting both the advancement brought by technology and what is ethical (27).

## 6 Conclusion and future work

A PoA consensus algorithm-driven blockchain-based framework was introduced for observing and tracking healthcare industry assets. It operates using the ERC-20 protocol. The tests demonstrated that the framework is easy to use and very effective. In Figure 7, we can see a statistical chart that clearly demonstrates many POA-confirmed transactions carried out successfully in the framework. It proves that the framework can guarantee that asset records are accurate and real. Among the transactions analyzed after three rounds of POA consensus validation, only a tiny number were marked as potential threats. Therefore, it is important to carry out more research to uncover and solve any security issues that could threaten the system’s security. The novel system demonstrates that combining blockchain and PoA can work well. The asset monitoring and tracking framework on blockchain eliminates many weaknesses of current methods, saves costs and improves security, privacy and efficiency, as revealed through the strong success rate in completing verified transactions. Despite these advances, more work is needed in security, performance refinement and validation of the overall framework in hospitals. It is important to work on reinforcing the security of the framework moving forward. This requires analyzing advanced systems for logging in, safeguarding information and monitoring unusual activity to make the system more resistant to threats. Furthermore, better performance improves both the scalability and efficiency of the Simplified Framework. Further research may study Proof of Stake (28, 29) and sharding (30, 31) as possible solutions to increasing speed and reducing gas fees in blockchain. In addition, testing the framework in real healthcare situations helps confirm its usefulness and effectiveness. Partnering with healthcare organizations and testing the framework with demo users will help in assessing how it functions, impacts patients and fits within current healthcare systems.

## Data Availability

The original contributions presented in the study are included in the article/supplementary material, further inquiries can be directed to the corresponding author.

## Footnotes

1
https://etherscan.io/gastracker
